# Book Review: La paradoja de jugar en tríada. El juego motor en tríada

**DOI:** 10.3389/fpsyg.2020.612587

**Published:** 2020-12-03

**Authors:** Raúl Martínez-Santos

**Affiliations:** Faculty of Education and Sport, University of the Basque Country, Vitoria-Gasteiz, Spain

**Keywords:** traditional game, motor praxeology, physical education, relational ambivalence, paradoxical effects, sociomotricity

In the wake of Parlebas' analysis of the inner communication structures of sporting games (Parlebas, 1986), Pic and Navarro coproduce an intriguing book to extend our comprehension of the internal logic of ludomotor situations: *The paradox of triadic game-playing. The triadic motor games*. This work, very much indebted to the doctoral thesis of the first of the authors, brings to light the interaction possibilities that lie beyond the one-on-one, dyadic sporting duels and their unexpected, hard-to-deal-with praxic consequences. Let us admit, to start with, that we are considering here a highly technical, conceptually demanding text with a, severe prose that sometimes requires from the addressees an unusual commitment to carry on reading. However, this difficulty is clearly balanced throughout the text by the authors' passion for traditional games and physical education: “We are convinced that triadic motor games contain a hidden pedagogical message that we must learn to appreciate. Playing triadic games means learning a form of paradoxical, rebalancing situations” (p. 9).

Pic and Navarro present a clearly structured, well-informed text on *ludic triads*: motor games in which three teams play against each other trying to outscore their opponents while not being defeated by the subtilities of the emerging paradoxes. The different academic viewpoints by which the study of triadic relationships is fuelled are presented in the first chapter, although all these lines of analysis (mathematical, social, cultural, and ludomotor ones) are secondary in regard to the works by the American sociologist Theodore Caplow on the dynamics of alliances and collaboration in two-against-one situations. The second chapter is specifically dedicated to the analysis of the mathematical properties of motor team-triads, which I would rather call *N-3 games* after Parlebas' classification of the networks of motor communication of sports: the identification of three main properties of the networks of these tagging games, namely, “circulation,” “transitivity,” and “interactivity,” allows the authors to complete a *census of triads* with 13 functional cases and many interesting nuances. The third, final chapter is dedicated to the empirical study of these games' “playability” and the emergence of paradoxical links in triadic tagging games from an educational point of view: the inclusion of a third competitive agent proves to be fruitful in tactical and relational decisions, and fertile in creativity. Moreover, an extra section contains the descriptions of 20 team games belonging to these 13 playable motor triads that help envisioning the complexity and richness of team motor triads.

However, what makes this book really valuable is the amount of occasions on which its readers will find their knowledge and beliefs challenged and disturbed. My main concern is that the authors have hardly succeeded in making a clear distinction between *pedagogical aims* (the alleged interest of working on ambivalent, paradoxical relationships), *educational means* (the fruitfulness of traditional games), and *structural analysis* (the communication roles and network properties of team triads). This may explain the lack of discussion of a major question: Why are team triads so scarce in traditional games? This question, the grounds on which they build their passionate research actually, is what justifies in fact the whole project: A sort of ludomotor engineering that tweaks and puts tradition to the test in a methodical, sophisticated way. In section 2.2, for instance, they strive to explain that a triad is always something more than a dyad, however its structural properties or the interpretations of the players. But the example they propose as a sort of transition between a dyad and a triad, the game called The Bear and its guardian, must be severely transformed to be so, and the original Parlebasian meaning of “sociomotor role” apparently reconsidered. There is a chance that this question requires a clearer distinction of the strategical, social roles proposed by Caplow, and the rules-based roles of game-players, who can be different within the same team (i.e., fielder and goalie in soccer; pitcher, catcher, and fielder in baseball, etc.).

Pic and Navarro seem to think of triadic games as an evolution of dyads. To them, triadic social systems seem to be an advanced option, and evolutional possibility. But the starting point could be just the opposite: Monodic or dyadic action systems could be seen as diminished, reduced social systems in which the triadic semiotic nature of reality cannot be fulfilled in terms of motor interaction. Any motor situation in which more than three agents interact leads to triadic motor interaction systems, that is, to motor action systems in which one player's acts must be interpreted as positive or negative in relation to any other two players' relationship. In team sports one's opponent's opponent is always a partner, and one's partner's opponent is always an opponent, but in Sitting ball this logic does not apply because the rules of the game do not enforce so, despite the fact that players in these ambivalent games are as constrained by the rules as players in team duels. Any paradoxical property of the internal logic of N-3 games is as much caused by the rules as its absence in sports, for any ludic paradox is a *praxic consequence* of rational motor decisions that break rationality while playing by the rules. Is there anything more absurd than being penalized by one's own scoring merits?

As I said above, this book has the ability to challenge one's beliefs. In my case, it turns upside down my childhood experience of playing tags and what I believed they are and can be used for. Pic and Navarros tagging triads break up with the socio-affective logic of ambivalent-unstable traditional games in which role-changing semiotricity leads to personal preferences rather than strategic choices. Furthermore, the inclusion of scoring artifacts like hats and cloths to fill in *structural holes* of the motor interaction networks of these games makes me wonder how close these team-triads can get to the logic of sports competition before losing their playful nature. Another paradox we must be aware of.

*The paradox of triadic game-playing* is a remarkable contribution to the field of motor praxeology because the authors openly aim to connect socially and educationally relevant topics with the inner, objective characteristics of a coherent type of motor tasks. Pic and Navarro have risked to take Parlebas' baton and successfully explored a subset of sociomotor networks from the rules, but beyond the duels. I feel as thankful for their contribution as intrepid to prompt them to considerer a second edition in which the physical educationist's point of view prevail in the presentation of the concepts, the development of the models, the description of the games, and the highlighting of the countless treasures that this *unorthodox* piece of work contains. I look forward to it!

## Author Contributions

The author confirms being the sole contributor of this work and has approved it for publication.

## Conflict of Interest

The author declares that the research was conducted in the absence of any commercial or financial relationships that could be construed as a potential conflict of interest.

## References

[B1] Parlebas P. (1986). Éléments de Sociologie du Sport. Paris: PUF.

